# Different Post-Sowing Nitrogen Management Approaches Required to Improve Nitrogen and Water Use Efficiency of Canola and Mustard

**DOI:** 10.3389/fpls.2020.01111

**Published:** 2020-07-21

**Authors:** Amritbir Riar, Gurjeet Gill, Glenn McDonald

**Affiliations:** ^1^ School of Agriculture Food and Wine, The University of Adelaide, Waite Campus, Adelaide, SA, Australia; ^2^ Research Institute of Organic Agriculture (FiBL), Frick, Switzerland

**Keywords:** nitrogen, water use, water distribution, N use efficiency, water use efficiency

## Abstract

Strategic use of nitrogen (N) may improve N use efficiency, but there is limited information on the influence of N supply at crucial growth stages on N accumulation, water use, and water use efficiency of canola and mustard. In this study, we hypothesize that genetic variation among canola and mustard can alter the response of timing and rate of post-sowing N application at targeted phenological growth stages by improving N and water use and their efficiencies. Field experiments were conducted in South Australia during two growing seasons with contrasting water availabilities. Two mustard and four canola cultivars, including two triazine tolerant (TT) and two non-TT cultivars were evaluated under different post-sowing N application strategies comprising three N rates and different timings of application. Mustard used more water than canola in the season with higher rainfall, but canola and mustard used similar amounts of water in the drier season. Nitrogen increased the water use efficiency (WUE) of canola and mustard cultivars. Nitrogen rate and timing did not influence the total water use of canola and mustard but influenced the partitioning of pre- and post-flowering water use. Even though, highest N uptake was observed in the treatment with continuous supply of N with 200 kg N ha^−1^ in five splits it did not influence the N efficiencies parameters which indicate that yield of canola and mustard are limited by N rate in these environments. In treatment with limited N supply, targeting N at the rosette stage improve N use efficiency of canola and mustard. However, the limited N uptake potential of mustard makes timing of N application the most important consideration whereas correct N rate should be main consideration for canola.

## Introduction

Canola (*Brassica napus* L. cv.) and mustard (*B. juncea*) together are the third-largest oilseed crop globally, with production of 75 MT yr^−1^ on over 35 million ha in 2018 (FAO, 2018). Canola products have high value for high protein meal for livestock, unsaturated oil for human consumption and also as biofuel. Plant breeding and agronomy have improved the adaptation and productivity of canola and mustard in Australia (Kirkegaard et al., 2016; Meier et al., 2020) resulting in an improvement in average yields from 0.5 t ha^−1^ in 1961 to 2.0 t ha^−1^ in 2018 (FAO, 2018). Canola has become a profitable crop as well as contributing to weed control and disease break for cereals in cropping systems around the globe (Heap, 2014; Kirkegaard et al., 2016). Production has also increased significantly in Australia over the last decade from c. 1.8 Mt in 2008–2009 to 3.9 Mt in 2017–2019 (ABARES, 2020). However, most of this increase has come from an expansion of area rather an increase in mean yield, which has shown relatively little change (ABARES, 2020).

Australian production of canola is based on both open-pollinated (genetically “true to type” OP) and hybrid varieties (F1) hybrids, and both conventional and triazine-tolerant (TT) varieties are grown (Brill et al., 2016; Kirkegaard et al., 2016; Zhang et al., 2016). Mustard is a minor crop, but it is suited to dry areas, and canola-quality *B. juncea* that meets Australian oilseed industry standards are available (Burton et al., 2008). Open-pollinated TT varieties are currently the main varieties grown in Australia (Kirkegaard et al., 2016), but imidazolinone herbicide-tolerant canola cultivars are becoming popular due to their higher yield potential (Hudson and Richards, 2014).

Average yields of canola in Australia are generally below their water-limited potential (Kirkegaard et al., 2016). Canola and mustard have higher demands for N, but lower water and N use efficiency (NUE) compared to cereals (Hocking et al., 1997a; Hocking et al., 1997b; Dreccer et al., 2000). In rainfed environments, N and water interact strongly to determine yield and poor uptake and use of one can lead to inefficient use of the other resource (Sadras, 2004; Ma and Herath, 2016; Riar et al., 2016). Inefficient use of water and N may be partly responsible for the sub-optimal performance of canola. Maaz et al. (2016) showed that greater water availability could improve N uptake and utilization efficiency of canola. To achieve the full yield potential of canola and mustard, it is necessary to overcome constraints limiting their growth and production in such environments. It will be challenging to realize the full benefits of genetic improvement without improving the N and water use of these crops (Sinclair and Rufty, 2012). Understanding the response to N availability at various growth stages in improving N and water use of canola and mustard could play an essential role in enhancing productivity in water-limited environments with low soil fertility.

Nitrogen is expensive and a difficult to manage input in environments with variable rainfall. Nitrogen losses from agricultural systems are also becoming a serious concern. Recovery of fertilizer N in crops is generally less than 50% (Fageria and Baligar, 2005), which cannot be justified from environmental and economic perspectives (Grant et al., 2002). Campbell et al. (2004) stated that total N yield is a function of plant-available water as water is a significant driver of grain yield in rainfed systems. Moreover, water deficits at a critical growth stage can limit N uptake and utilization in plants (Benjamin et al., 1997) and can reduce crop response to N fertilizers. Many studies have shown the importance of the effects of water availability on N response of crops and vice-versa (Sadras, 2004; Norton and Wachsmann, 2006; Sinclair and Rufty, 2012; Pan et al., 2016). According to Riar et al. (2016) the gap between actual and potential yield in rainfed system was lower when water and N equally co-limited the growth of canola.

In rainfed environments, water is a limiting resource, and its availability depends on the amount of water stored during the fallow and the amount of growing season rainfall. It is widely accepted that the management of fertilizer inputs is one of the essential tools for the improvement of WUE in these environments (Cooper et al., 1987; Angus and Van Herwaarden, 2001; Sadras and Roget, 2004). Many previous studies on the water use of oilseed crops have not investigated the influence of N on water uptake from different depths in the soil profile (Johnston et al., 2002; Campbell et al., 2007; Angadi et al., 2008; Gan et al., 2009). A common practice in N management in canola is to apply N as split or delayed applications to match N supply with crop demand better (Norton, 2016). The effects of N applications at targeted growth stages on the growth and seed yield of canola and mustard were reported in an earlier study (Riar et al., 2017). In the study reported here, we tested the hypothesis that targeting post sowing N application to specific phenological growth stages can improve NUE and WUE in canola and mustard. Still, responses may differ due to genetic variation associated with different yield potentials of species and among cultivars within species. Six different canola and mustard varieties, representative of the range of varieties grown in southern-Australia were compared to examine whether genetic differences in yield potential affect the responses to N and are reflected in differences in patterns of water and N uptake and efficiency.

## Materials and Methods

### Site Description

**Figure 1:**
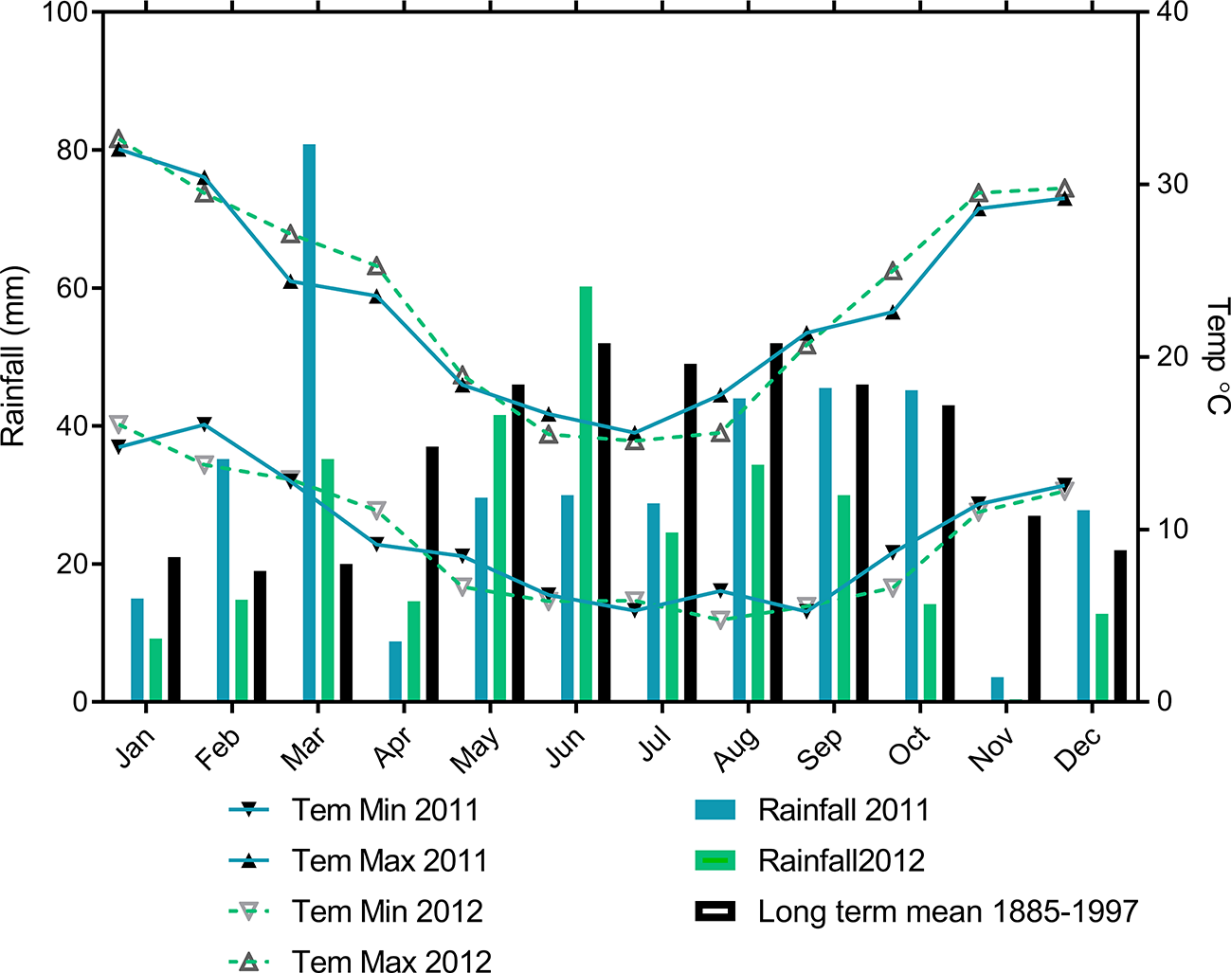
Monthly mean maximum and minimum temperature, monthly rainfall during 2011 and 2012 with long-term means for Roseworthy.

Field experiments were conducted at the Roseworthy farm of the University of Adelaide (latitude 34.53°S; longitude 138.72°E), South Australia during the 2011 and 2012 growing seasons. The long term annual average rainfall for Roseworthy is 440 mm with a growing season average rainfall (defined in South Australia as rainfall from April to October (French and Schultz, 1984)) of 329 mm rainfall (**Figure 1**).

The main soil type of the sites in 2011 and 2012 was classified as calcisols (FAO, 1988; Driessen et al., 2000), equivalent to calcarosol in the Australian soil classification (Isbell, 2002). To estimate the average soil moisture and nitrate-N in each experiment, nine soil cores were taken to a depth of 100 cm 1 day prior to sowing using a 4 cm hydraulic core. The soil cores were divided into 20 cm increments, bulked, dried at 40°C, and sieved (<2 mm) for analysis by a commercial laboratory (CSBP Perth, Western Australia). The amount of the mineral-N (ammonium + nitrate) in the 0–100 cm layer was 77 and 71 kg ha^−1^ in 2011 and 2012, respectively. Detailed soil characteristics of the experimental sites are given in **Table 1**.

**Table 1:** Soil characteristics of each site used during 2011 and 2012.

Year	Layer (cm)	Ammonium N^1^ (mg kg^−1^)	Nitrate N^2^ (mg kg^−1^)	Colwell P^3^ (mg kg^−1^)	Colwell K^4^ (mg kg^−1^)	Sulfur^5^ (mg kg^−1^)	Organic C^6^ (%)	Conductivity^7^ (dS/m)	pH level H_2_O^8^ (pH)	Boron^9^ (mg kg^−1^)	Bulk Density
2011	0–20	7.0	4.0	23.0	580	7.2	0.99	0.211	7.5	2.07	1.31
	20–40	3.0	2.0	7.0	220	7.2	0.57	0.170	8.2	2.39	1.21
	40–60	2.0	1.0	10.0	222	18.0	0.32	0.230	8.1	6.18	1.24
	60–80	2.0	2.0	3.0	424	56.8	0.20	0.464	8.5	17.03	1.41
	80–100	1.0	5.0	<2.0	543	66.7	0.15	0.404	8.7	25.50	1.41
2012	0–20	12.7	10	56.7	509	20.1	1.21	0.300	7.7	1.89	1.31
	20–40	5.3	4.0	13.7	192	12.5	0.66	0.200	8.6	3.25	1.23
	40–60	4.0	1.6	9.7	132	16.8	0.34	0.210	8.8	4.21	1.36
	60–80	3.0	1.7	5.0	179	29.6	0.25	0.428	9.1	9.62	1.33
	80–100	2.3	2.0	3.0	276	55.0	0.19	0.622	9.3	13.19	1.13

^1^&^2^ 2M KCL solution for 1 hour; ^3^Bicarbonate-extractable P; ^4^Bicarbonate-extractable K; ^5^KCI-40; ^6^Organic carbon (Walkley and Black 1934). ^7&8^1:5 soil-water extract. ^9^1:4 dilute HCL.

Analyses were conducted by CSBP Soil and Plant Analysis Laboratories, Perth WA using the methods described in Rayment and Lyons (Rayment and Lyons, 2011).

### Experimental Design and Crop Management

Two mustard (Varuna and Oasis) and four canola cultivars with mid-maturity, including two triazine-tolerant [TT—Fighter TT (OP) and Hyola555TT (hybrid) and two non-TT cultivars (AV Garnet (OP) and Hyola575cl (Imidazolinone herbicide-tolerant canola cultivar)], were evaluated under different N application strategies, comprising three N rates (0, 100, and 200 kg N ha^−1^ as granular urea; 46% N) and different timings of application (**Table 2**). In 2011, the effect of two times of sowing was investigated, and the treatments were arranged in a split-split plot design with time of sowing as the main-plots, canola, and mustard cultivar as the sub-plots and N treatments as sub-sub-plots in three replications. In 2011, the effect of time of sowing on seed yield was not significant (Riar et al., 2017) so in 2012, only one time of sowing was used, and treatments were arranged in a split-plot design with cultivars in main-plots and N treatments in sub-plots with three replications. In 2011, N and water use and efficiencies were only measured in the first time of sowing.

**Table 2:** Details of N rates, number of split applications and growth stages in the different N treatments used for canola and mustard during 2011 and 2012 (BBCH-scale (canola) where GS30, Beginning of stem elongation: no internodes (“rosette”); GS51, Flower buds visible from above (“green bud”); 63, 30% of flowers on main raceme open (“flowering”); 67, Flowering declining: majority of petals fallen(“pod initiation”); 71, 10% of pods have reached final size (“pod development”) (Lancashire et al., 1991).

Year	N Rate	Splits	Applied N kg ha^−1^ at different growth stages (BBCH scale)				
			Rosette (GS30)	Green bud (GS51)	Flowering (GS63)	Pod initiation (GS67)	Pod development (GS71)
2011 + 2012	0	0					
2011	100	3	33.3		33.3		33.3
2011	100	2		50		50	
2011	100	2			50		50
2012	100	1	100				
2012	100	1		100			
2012	100	1			100		
2012	100	5	20	20	20	20	20
2011 + 2012	200	5	40	40	40	40	40

The trials were sown with a cone seeder with knifepoint drill and press wheels at a depth of 25 mm. Plot area was 15 m^2^, with each plot being 10 meters long by six rows with a 250 mm inter-row spacing. Basal fertilizers were 10 kg N ha^−1^, and 11 kg P ha^−1^ as diammonium phosphate (DAP) applied at sowing and 100 kg S ha^−1^ as a pre-planting gypsum application. Seeding rates were adjusted for each cultivar based on their seed weight and germination percentage to achieve a plant establishment of 35 plants m^−2^. Plant numbers were counted after crop establishment, and it showed that on average there was 84% and 82% establishment in 2011 and 2012, respectively. Weeds and diseases were managed with standard agronomic practices, and overall weed and disease incidence was minimal during both years.

Nitrogen treatments were designed to generate a range in crop biomass and canopy size (**Table 2**) and targeted at specific growth stages. A control treatment with no N (N0) and a high N control (N200) in which a total of 200 kg N ha^−1^ was applied in five equal split applications at key growth stages: rosette (GS30), green-bud (GS51), 30% of flowers on main raceme open (GS61), start of pod filling (GS67), and 10% pod maturity (GS71) was used to maintain a steady supply of N throughout the season with the aim of having a non-limiting supply of N. The growth stages were recorded using the BBCH canola scale (Lancashire et al., 1991). The low and high N controls were designed to provide a boundary function of crop response to N in both years. All other treatments with 100 kg N ha^−1^ were designed to examine the effects of N supply at a specific growth stage on water and N use and their efficiencies. In South Australian rainfed farming systems, N is generally applied prior to sowing or a maximum 10 kg ha^−1^ at sowing and remainder top-dressed after emergence (Parker et al., 2009), as applying the entire N at the start of the season can be economically risky because of variable spring rainfall, and the risk of high rates of N reducing seedling emergence if it applied near the seed. Where a split application was used, the rates of N were equal at all times of applications. Nitrogen (granular urea; 46% N) was broadcast using a hand spreader (Scotts Easy Handheld Spreader) at the desired growth stage either when the soil was wet or if rainfall was forecast within 24 h after application. On average, there was 13.2 and 9.92 mm rainfall in the week following the N applications in 2011 and 2012 respectively.

### Sampling and Measurements

Destructive samples from two rows of 50 cm length (0.25 m^2^) were taken at flowering and maturity to measure crop biomass. Leaf loss at maturity was not accounted for in the dry matter and N measurements at harvest. Nitrogen content of shoots was measured at 50% flowering (GS65) and maturity (GS99). For each sampling, a quadrat sample (two rows of 50 cm length) was harvested and samples were dried in an oven at 80°C for 48 h. A weight-based subsample equivalent to 25% of the quadrat sample were analyzed for N content and then converted to N uptake m^−2^ accordingly.

The dried samples were ground through a 2 mm sieve prior to chemical analysis. Nitrogen concentrations of the total above-ground biomass samples at flowering and maturity (pods + seed + straw) were measured with a LECO combustion analyzer (NitroFlow 60, St. Joseph, Michigan.); the ground plant samples were combusted at 950˚C and flushed with oxygen and the N oxides measured. Nitrogen concentration in whole seed was measured using a near infra-red grain analyzer (Cropscan 1000B, Next Instruments Pty Ltd). Nitrogen uptake was calculated from the whole plant N concentration and the crop biomass at the time of sampling. Nitrogen harvest index (NHI) was calculated from the seed N content and the total N uptake at maturity:

(1) $$\text{NHI}=\frac{\text{Total N uptake in seed}}{\text{Total N uptake in above-ground biomass}}$$

The efficiency of N use for canola and mustard was calculated by using the following formulae (Fageria and Baligar, 2005; Rathke et al., 2006):

(2) $$\begin{array}{c} \;{\text{NUE}}_{\mathrm{SY}}\;(\text{kg k}{\text{g}}^{-1})\\ =\frac{\text{Seed yield}}{\text{Total mineral N}\;+\;\text{fertliser N}\;+\;\text{N mineralization}} \end{array}$$

where growing season mineralized N was estimated by using the following equation proposed by Dunsford et al. (2015):

(3) N mineralization=Growing Season Rainfall x 0.15 x Organic matter Content %

(4) $$\text{Nuptake efficiency}\;(\text{kg k}{\text{g}}^{-1})=\frac{\mathrm{Total N uptake}}{\text{Total mineral N}+\text{fertiliser N}+\text{N mineralisation}}$$

(5) $$\text{Agronomic efficiency}\;(\text{kg k}{\text{g}}^{-1})=\frac{{\text{S}}_{\text{F}}-{\text{S}}_{\text{C}}}{\text{F}}$$

(6) $$\text{Apparent Recovery}\;(\%)=\frac{{\text{N}}_{\text{F}}-{\text{N}}_{\text{C}}}{\text{F}}\times 100$$

(7) $$\text{Physiological efficiency }\;(\text{kg k}{\text{g}}^{-1})=\frac{{\text{Y}}_{\text{F}}-{\text{Y}}_{\text{C}}}{{\text{N}}_{\text{F}}-{\text{N}}_{\text{C}}}$$

where S_F_ and S_C_ are the seed yield of the fertilized and unfertilized plots, Y_F_ and Y_C_ are the total above-ground biomass of the fertilized and unfertilized plots, N_F_ and N_C_ is the N contained in biological yield (kg ha^−1^) of fertilized and unfertilized plots, and F was the amount of fertilizer N applied as granular urea (McDonald, 1989; Fageria and Baligar, 2005). Agronomic efficiency reflects the efficiency with which applied N is used, and physiological efficiency can be viewed as the response of crop to additional N uptake from fertilizer. The total soil N measure at the start of the seasons was used to estimate N supply including seasonal N mineralization estimate.

Water use and WUE were measured in selected treatments with total N rates of 0, 100, and 200 kg N ha^−1^. In both years WUE was measured in 0 kg N ha^−1^ control and 200 kg N ha^−1^ in five equal splits. In 2011, measurements were also made in 100 kg N ha^−1^ in three equal splits at rosette (GS30), flowering (GS63), and pod development (GS71) and in 2012, in the treatment with a single application of 100 kg N ha^−1^ at rosette (GS30). To examine genetic variation in transpiration efficiency (TE) in 2011 the C isotope discrimination based on the relative abundance of the stable isotopes ^12^C and ^13^C was measured using mass spectrometry (elemental analyzer, E A1108, Series1: Carlo Erba Istrumentazione, Milan, Italy). Measurements were based on leaves collected from four randomly-selected plants per plot at the rosette stage in the nil N control plots. The leaf material was dried at 80°C for 48 h, ground twice and the second time in a ball mill before the isotopic composition was measured. Carbon isotope composition values (δ ^13^C) were converted to Δ by assuming isotopic composition of air to −8 ‰. Soil moisture content was measured to 100 cm depth pre-sowing, at flowering and maturity using a 4 cm hydraulic core. A single core was taken from each plot across all replicates at each time of sampling. Cores were sub-divided into five layers each of 20 cm length to assess the crop water extraction from various depths. The change in soil water over 0–100 cm was used to estimate crop water use (CWU) in treatments with 0, 100, and 200 kg N^−1^ and assuming no drainage below the root zone, where P is growing season rainfall, ΔS is the difference in soil moisture between harvest and sowing:

(8) CWU (mm)=P-ΔS

In addition, water use of two canola cultivars with contrasting early vigor (AV Garnet and FighterTT) was measured with a capacitance probe (Diviner 2000, Sentek. SA) at 16 different times during the growing season.

### Statistical Analysis

The data were analyzed by analysis of variance (ANOVA) using the GenStat statistical analysis software (15th edition; VSN International) (VSN International, 2013). In the model, cultivar and N treatments were considered as fixed effects and replicates as a random effect. A combined analysis of the 2 years was not possible because of the different N treatments in the two experiments (**Table 2**), but an analysis based on the treatments common to both years (0 and 200 kg N ha^−1^) was conducted in which year was considered as a random effect. This analysis showed significant interactions with years for water use and N uptake, so the results for each year are presented. Orthogonal comparisons were used to compare the different groups of cultivars and N treatment based on a single degree of freedom comparisons. For other statistical comparisons, Tukey *post hoc* test Honest Significant Differences (HSD) at 5% level of probability (P < 0.05) was used to compare the treatments.

## Results

Growing season (April-October) rainfall was similar in both years (232 mm in 2011 and 220 mm in 2012) (**Table 3**), but the annual rainfall (January-December) was 102 mm lower in 2012 (292 mm) than 2011 (394 mm), and the distribution of rainfall varied considerably between the 2 years. In 2011, the season had a wet start with above-average rainfall in February and March, so the soil water to a one-meter depth at sowing was 230 mm whereas 2012 had a dry start with starting soil water of 70 mm. In 2012, rainfall during the spring (September–November) was less than half of that received over this period in 2011 (**Table 3**). From here on, we consider 2011 as an average year (462 mm soil water during crop growing period (sowing to harvest rainfall+ Soil water at sowing) and 2012 as a drier than average year (290 mm soil water during crop growing period (sowing to harvest rainfall + soil water at sowing) (**Table 3**).

**Table 3:** Rainfall and water availabilities at various phases of two growing years (2011 and 2012).

Year	Annual rainfall	GS rainfall	Pre-sowing rainfall (Jan-April)	Pre-flowering rainfall (May-August)	Post-flowering rainfall (Sept-Nov)	Soil water at sowing
2011	394	232	140	132	94	230
2012	292	220	74	161	45	70
Long term average	440	329	98	202	118	

### Water Use Pattern and Efficiency

#### Water Distribution in the Soil Profile

**Figure 2:**
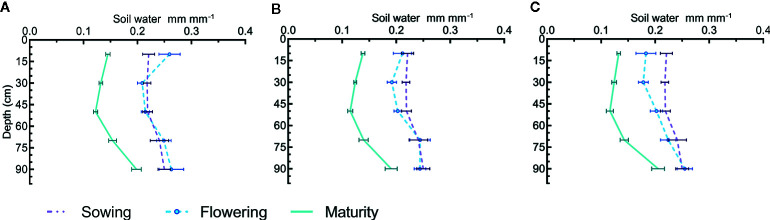
Soil water distribution in profile at sowing, flowering and maturity for canola and mustard cultivars under three different N regimes; **(A)** 0, **(B)** 100, and **(C)** 200 kg N ha^−1^ in 2011. Where horizontal bars show the standard error for the measured value. Cultivars did not differ in water extraction pattern so only main effects of N are shown as cultivar × N interactions were also not significant.

As a result of high pre-sowing rainfall in 2011, the soil water content was >0.2 mm mm^−1^ at all depths at the time of sowing (**Figure 2**). Analysis of different depth increments for water content at flowering showed that cultivars did not differ in water extraction at any depth. However, a significant difference was observed in water extraction at 0–20 cm for N treatments at flowering (P = 0.029) and maturity (P = 0.022). The reduction in soil water increased as the rate of N increased (**Figure 2**). There were no changes in soil profile water content at flowering in treatments with no N applied and the reduction with 100 kg N ha^−1^ was smaller than measured with 200 kg N ha^−1^ (**Figures 2A, B**).

**Figure 3:**
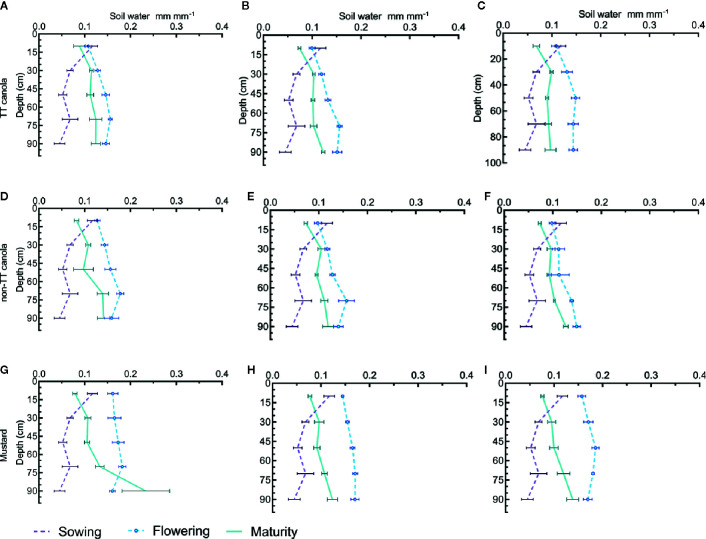
Soil water distribution in profile at sowing, flowering and maturity for TT canola cultivars (Fighter and Hyola555TT) **(A–C)**, non-TT canola cultivars (AV Garnet and Hyola 575cl) **(D–F)**, and mustard cultivars (Oasis and Varuna) **(G–I)** at 0 kg N ha^−1^ **(A, D, G)** 100 kg N ha^−1^ **(B, E, H)**, and 200 kg N ha^−1^ **(C, F, I)** in 2012. Where horizontal bars show the standard error for the measured value.

In contrast to 2011, the very dry summer and autumn in 2012 resulted in the soil profile at sowing being very dry (**Figure 3**) however, not all rainfall received until flowering was used by canola (**Figures 3A–F**) so there was some accumulation of soil water but soil moisture at flowering was still generally lower than at flowering in 2011. At maturity, canola (both TT and non-TT) and mustard were able to dry the soil profile between flowering and maturity. The reduction in soil moisture increased with N rate and ended to be greater in mustard than in canola.

#### Pre-Flowering and Post-Flowering Water Use

**Figure 4:**
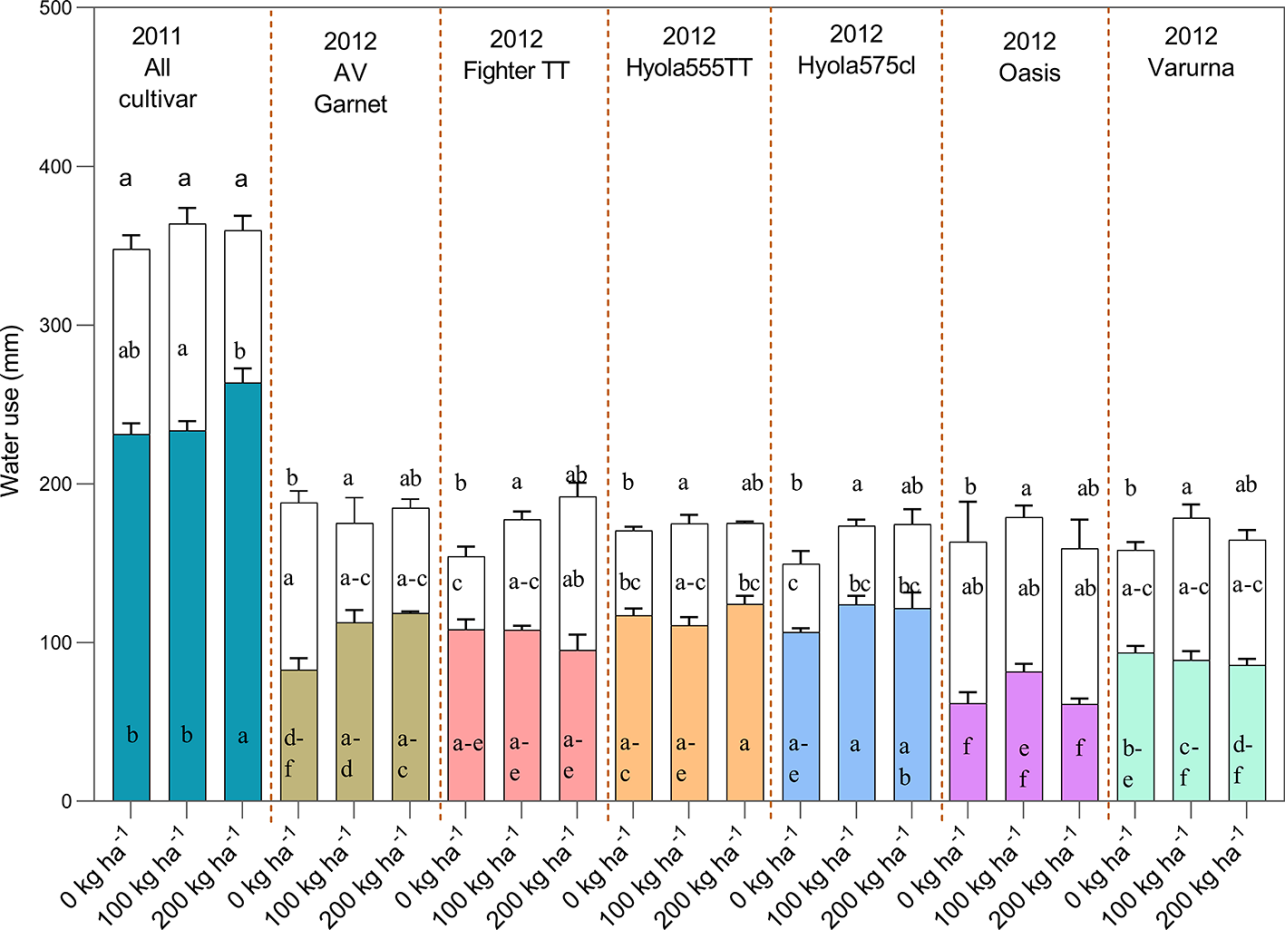
Total water use [pre (colored bars) and post-flowering water use (transparent bars)] of different cultivars as influenced by N rates of 0, 100, and 200 kg N ha^−1^ during the growing season of 2011 and 2012 (The 200 kg N ha^−1^ treatment had only received 80 kg N ha^−1^ by flowering). In 2011 only mean of all cultivar for N treatments are shown as only N treatments shown significant effects whereas in 2012 the cultivar × N interaction was significant. Bars showing same or overlapping letters do not exhibit significant difference among each other.

Crop water use by canola and mustard cultivars were not significantly different in each year. On average, CWU by all cultivars was 349 ± 2.9 mm in 2011 and 171 ± 2.3 mm in 2012 (**Figure 4**). There was no significant difference in CWU by mustard and canola in either year, but the pattern of water use differed, which was more strongly expressed in 2012 (**Table 4** and **Figure 4**). While there was no difference in total water use between canola and mustard, there was a tendency for mustard to use less water than canola during the pre-flowering period [3.7% less in 2011 (NS); 29% in 2012 (*P* <.0001)] but more water than canola in the post-flowering period [14% more in 2011 (*P*=0.015); 26% in 2012 (*P*= 0.0001)] (**Table 4**). There was no significant difference in CWU between TT varieties and non-TT varieties or between open-pollinated and hybrid varieties in 2011 (**Table 4**). In 2012 the pattern of water use between open-pollinated and hybrid varieties differed significantly: open-pollinated varieties used significantly less water (14 mm) than hybrid varieties up to flowering and 22 mm more water after flowering (**Figure 4**).

**Table 4:** The significance of single degree of freedom orthogonal comparisons for total, pre, and post-flowering water use and water use efficiency of different cultivars are shown: canola vs mustard TT canola vs non-TT canola (TT vs non-TT), and open-pollinated canola vs hybrid canola (OPc vs HYc).

Orthogonal comparisons		Water use (mm)			WUE (kg ha^−1^ mm^−1^)	
		Pre-Flowering	Post-Flowering	Total	GY	DM
2011						
Canola vs Mustard		NS	0.015	NS	NS	0.0186
	TT vs nonTT	NS	NS	NS	NS	<0.001
	OPc vs HYc	NS	NS	NS	NS	NS
2012						
Canola vs Mustard		<0.001	<0.0001	NS	0.0106	NS
	TT vs nonTT	NS	NS	NS	0.0034	0.0339
	OPc vs HYc	<0.0003	0.0002	NS	0.0057	0.0018

A lack of differences between single degree of freedom orthogonal comparisons is noted by NS (P > 0.05 = not significant).

In 2011, N treatments affected the partitioning of water use between pre-flowering and post-flowering growth periods without changing the total water use of canola and mustard (**Figure 4**). All cultivars with a total application of 200 kg N ha^−1^ split between five key growth stages used more water than 100 kg N ha^−1^ in three splits and the control prior to flowering (**Figure 4**).

In 2012, the pattern of pre-flowering and post-flowering water use was similar to 2011 in canola and mustard cultivars (**Figure 4**). However, there was a significant cultivar × nitrogen interaction for total crop water use. In general, pre and post-flowering water use was higher in treatments with N as compared to the control.

In addition, water use of two canola cultivars with contrasting early vigor (AV Garnet and FighterTT) revealed that water use of these two cultivars was not significantly different at any sampling time. Crop water use in treatments supplied with N was more than the control, but the difference was not significant. Total water used by the crop was similar between N treatments and the control. Water was extracted from the soil profile at a depth of 40 cm was evident, but the 40–60 cm profile depth mostly remained unchanged during crop growth and some soil water accumulated during the growing season in the depth of 70–100 cm.

#### Water Use Efficiency (WUE)

**Figure 5:**
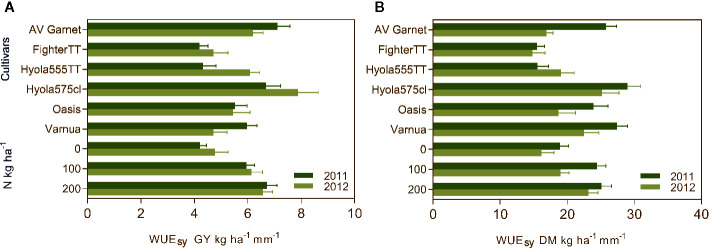
Water use efficiency grain yield (WUE_SY_) **(A)** and WUE dry matter (WUE_DM_) **(B)** of different cultivars under N rates of 0 kg N ha^−1^, 100 kg N ha^−1^, and 200 kg N ha^−1^ during the growing season of 2011 and 2012. Values are presented as mean of replications for cultivars and N treatments as cultivar × N treatment interactions for WUE_GY_ and WUE_DM_ were absent during both years.

Measurements of C isotope discrimination revealed Δ ranged from 18.74 ‰ to 19.79 ‰ but there were no significant genetic differences in stable carbon ratios among the cultivars (**Table S1**). Orthogonal contrasts indicated the mean WUE_SY_ and WUE_DM_ of canola and mustard were not significantly different in 2011 and 2012 (**Table 4** and **Figures 5A, B**), on an average WUE_SY_ was 5.7 kg ha^−1^ mm^−1^ in 2011 and 5.8 kg ha^−1^ mm^−1^ in 2012. However, in both years TT cultivars of canola had significantly lower WUE_SY_ (4.7 kg ha^−1^ mm^−1^ in 2011 and 5.4 kg ha^−1^ mm^−1^ in 2012) than non-TT cultivars (6.8 kg ha^−1^ mm^−1^ in 2011 and 7.0 kg ha^−1^ mm^−1^ in 2012) with Fighter TT having the lowest WUE in both seasons (**Table 4** and **Figure 3A**). The WUE_DM_ of non-TT cultivars was 26.7 kg ha^−1^ mm^−1^ and 21.1 kg ha^−1^ mm^−1^ compared to 16.7 kg ha^−1^ mm^−1^ and 16.4 kg ha^−1^ mm^−1^ for the TT cultivars in 2011 and 2012, respectively. The differences in WUE_SY_ and WUE_DM_ between the OP and hybrid cultivars was not significant in 2011, but the WUE_SY_ of the OP cultivar was 1.5 kg ha^−1^ mm^−1^, and WUE_DM_ was 6.3 kg ha^−1^ mm^−1^ lower than hybrid cultivars in 2012.

Applying fertilizer N improved WUE_SY_ in both years (**Figure 5A**). The highest WUE occurred when the crops received a regular supply of N throughout the season i.e. 200 kg N ha^−1^ in five splits. In 2011, the highest WUE_SY_ was achieved with 200 kg N ha^−1^ in five splits, followed by 100 kg N ha^−1^ in three splits and control (**Figure 5A**). In 2012, the highest WUE_SY_ was achieved with 200 kg N ha^−1^ in five splits followed by 100 kg N ha^−1^ at rosette and control, respectively (**Figure 5A**). Trends for WUE_DM_ were in same order as WUE_SY_. In 2011, WUE_DM_ did not differ between 200 kg N ha^−1^ and the split application of 100 kg N ha^−1^ (three splits starting at the rosette stage in 2011 and single dose at rosette stage in 2012), but both were higher than the WUE_DM_ in control (**Figure 5B**).

### Nitrogen Uptake and Use Efficiency

#### Nitrogen Uptake, Seed N Content, and Nitrogen Harvest Index

Total N uptake at maturity varied considerably between the two growing seasons. On average, the total N uptake by the crop at maturity in 2012 was only 37.5% of the 2011 uptake. Canola and mustard did not differ in N uptake during the pre- and post-flowering periods in 2011 (**Table 5**). Nitrogen uptake remained low in TT cultivars during the pre- and post-flowering growing periods; hence total N uptake in TT canola at maturity was lower than that of non-TT cultivars.

**Table 5:** N uptake and Nitrogen Harvest Index for different cultivars of canola and mustard during 2011 and 2012.

Cultivar	N uptake (kg ha^−1^)						NHI	
	Pre-flowering		Post-Flowering		Total			
	2011	2012	2011	2012	2011	2012	2011	2012
AV-Garnet	110^ab^	76^b^	92^a^	−11^a^	202^ab^	66^abc^	0.25^b^	0.47^a^
Fighter TT	89^ab^	86^ab^	62^a^	−27^a^	151^b^	59^c^	0.33^a^	0.44^a^
Hyola555TT	84^b^	109^a^	68^a^	−28^a^	152^ab^	81^ab^	0.27^ab^	0.40^a^
Hyola575cl	116^ab^	94^ab^	96^a^	−12^a^	211^a^	82^a^	0.26^b^	0.46^a^
Oasis	126^a^	69^b^	62^a^	−10^a^	188^ab^	60^bc^	0.13^c^	0.20^b^
Varuna	87^b^	79^ab^	113^a^	−12^a^	201^ab^	67^abc^	0.33^a^	0.49^a^
Orthogonal comparisons								
Canola vs Mustard	NS	0.028	NS	NS	NS	NS	0.034	0.003
TT vs nonTT	0.004	NS	NS	NS	0.003	NS	0.044	NS
OPc vs HYc	NS	0.025	NS	NS	NS	0.003	NS	NS

Contrasts were determined by Tukey test. Different lowercase letters indicate significant difference (Tukey's test P < 0.05). The significance of single degree of freedom orthogonal comparisons are also shown: canola vs mustard, TT canola vs non-TT canola (TT vs non-TT), and open-pollinated canola vs hybrid canola (OPc vs HYc). A lack of differences between single degree of freedom orthogonal comparisons is noted by NS (not significant).

Values are presented as mean of replications and mean of N treatments as there was no cultivar x N treatments interactions were found.

In 2012, total N uptake by TT and non-TT varieties was similar due to similar N uptake during the pre-flowering and post-flowering periods (**Table 5**). There was some reduction in shoot N during the post-flowering period in 2012, which varied from 10 to 28 kg N ha^−1^, which was due to loss of dry leaf matter. Total N uptake in open-pollinated cultivars was lower than the hybrid cultivars due to low N uptake during the pre-flowering period. Mustard had lower N uptake than canola during the pre-flowering period (P= 0.028), and the difference was not significant at maturity (P=0.076).

Nitrogen application improved the total N uptake in both years. Highest N uptake was achieved with the application of 200 kg N ha^−1^ in both years, followed by 100 kg N ha^−1^ and the control (**Tables 6** and **7**). Post-flowering N uptake was higher with 200 kg N ha^−1^ than all other N treatments in 2011. Nitrogen application at the rosette and green-bud stages in 2011 resulted in higher N uptake than the application of N at flowering. All other treatments that received 100 kg N ha^−1^ had similar total N uptake at maturity.

**Table 6:** N uptake, Nitrogen Harvest Index, and NUE of canola and mustard cultivars as under different N regimes during 2011.

N Rate	Targeted GS for N	N uptake kg ha^−1^			NHI	N uptake efficiency kg N kg^−1^ N supply	NUE_SY_	Agronomic efficiency kg kg^−1^ N fert.	Physiological efficiency Physiological efficiency kg kg ^-1^	Apparent recovery %
		Pre-flowering	Post-Flowering	Total						
0		70^c^	53^b^	123^c^	0.31^a^	1.33^a^	16.6^a^			
100	30,63, 71	122^a^	68^b^	189^b^	0.25^bc^	0.98^b^	11.3^b^	6.5^a^	45.0^a^	66.1^a^
100	51,67	110^ab^	88^ab^	198^ab^	0.22^c^	1.03^ab^	10.7^b^	5.2^a^	21.5^a^	74.9^a^
100	63,71	86^bc^	75^b^	161^bc^	0.24^bc^	0.84^b^	9.2^b^	2.4^a^	14.3^a^	37.9^a^
200	30,51,63,67,71	122^a^	127^a^	249^a^	0.28^ab^	0.85^b^	8.6^b^	4.8^a^	25.5^a^	62.7^a^
Orthogonal comparisons										
N vs no N		<.001	0.006	<.001	<.001	0.001	<.001			
100 kg N ha^−1^ vs 200 kg N ha^−1^		0.059	<.001	<.001	0.01	NS	NS	NS	NS	NS
100 R vs 100 GB/F		0.01	NS	NS	NS	NS	NS	0.013	<.001	NS
100 GB vs 100 F		0.019	NS	0.027	NS	NS	NS	0.023	NS	0.009

Growth stages were measured on BBCH-scale (canola): 30, Beginning of stem elongation: no internodes (“rosette”); 51, Flower buds visible from above (“green bud”); 63, 30% of flowers on main raceme open; 67, Flowering declining: majority of petals fallen and 71, 10% of pods have reached final size (Lancashire et al., 1991). Values are presented as mean of replications and mean of cultivars as there was no cultivar x N treatments interactions were found. Contrast were determined by Tukey test. Different lowercase letters indicate significant difference (Tukey's test P < 0.05). The significance of single degree of freedom orthogonal comparisons are also shown: N vs no N, 100 kg N ha^−1^ vs 200 kg N ha^−1^, 100 kg N ha^−1^ at rosette vs 100 kg N ha^−1^ at green-bud or Flowering (100 R vs 100 GB/F), and 100 kg N ha^−1^ at green-bud vs 100 kg N ha^−1^ at Flowering (100 GB vs 100 F). A lack of differences between single degree of freedom orthogonal comparisons is noted by NS (not significant).

**Table 7:** N uptake, Nitrogen Harvest Index, and NUE of canola and mustard cultivars under different N regimes during 2012.

N Rate	Targeted GS for N	N uptake kg ha^−1^			NHI	N uptake efficiency kg N kg^−1^ N supply	NUE_SY_	Agronomic efficiency kg kg^−1^ N fert.	Physiological efficiency kg kg ^-1^	Apparent recovery %
		Pre-flowering	Post-Flowering	Total						
0	0	57^c^	−8^a^	49^b^	0.41^ab^	0.37^a^	5.9^a^			
100	30	102^a^	−36^a^	65^b^	0.48^a^	0.28^ab^	4.6^b^	3.1^a^	44.2^a^	15.8^a^
100	51	99^ab^	−35^a^	64^b^	0.39^ab^	0.27^b^	3.6^bc^	1.0^a^	35.7^a^	15.0^a^
100	63	69^bc^	−4^a^	65^b^	0.44^ab^	0.28 ^ab^	4.1^bc^	2.0^a^	35.3^a^	15.7^a^
100	30,51,63,67,71	86^abc^	−16^a^	70^b^	0.36^b^	0.29^ab^	3.9^bc^	1.8^a^	38.3^a^	20.7^a^
200	30,51,63,67,71	101^a^	−1^a^	101^a^	0.38^ab^	0.30^ab^	3.4^c^	1.9^a^	39.6^a^	25.7^a^
Orthogonal comparisons										
N vs no N		<.001	NS	<.001	NS	NS	<.0001			
100 kg N ha^−1^ vs 200 kg N ha^−1^		NS	0.034	<.001	NS	NS	NS	NS	NS	0.092
100 S vs 100 SP		NS	NS	NS	0.008	NS	NS	NS	NS	NS
100 R vs 100 GB/F		0.061	NS	NS	0.037	NS	0.0267	0.014	NS	NS
100 GB vs 100 F		0.006	0.022	NS	NS	NS	NS	NS	NS	NS

Growth stages were measured on BBCH-scale (canola): 30, Beginning of stem elongation: no internodes (“rosette”); 51, Flower buds visible from above (“green bud”); 63, 30% of flowers on main raceme open; 67, Flowering declining: majority of petals fallen and 71, 10% of pods have reached final size (Lancashire et al., 1991). Values are presented as mean of replications and mean of cultivars as there was no cultivar x N treatments interactions were found. Contrast were determined by Tukey test. Different lowercase letters indicate significant difference (Tukey's test P < 0.05). The significance of single degree of freedom orthogonal comparisons are also shown: N vs no N, 100 kg N ha^−1^ vs 200 kg N ha^−1^, 100 kg N ha^−1^ at rosette vs 100 kg N ha^−1^ at green-bud or Flowering (100 R vs 100 GB/F), and 100 kg N ha^−1^ at green-bud vs 100 kg N ha^−1^ at Flowering (100 GB vs 100 F). A lack of differences between single degree of freedom orthogonal comparisons is noted by NS (not significant).

Difference in N uptake was not reflected in seed N content. Seed N content showed a significant cultivar × N interaction. On average, canola had 1.5%–3.0% and 2.3%–3.4% seed N content in 2011 and 2012, respectively compared to 1.1%–3.3% and 1.2%–4.1% in mustard in 2011 and 2012, respectively.

Nitrogen harvest index (NHI) of cultivars varied considerably between the seasons. In the average season (2011) with high N post-flowering uptake, NHI was lower (0.25) than the dry season (2012) with low total N post-flowering uptake (0.40) due to low water availability and also loss of N from leaf fall (**Table 5**). In both years, the lowest NHI was in Oasis (canola quality mustard) whereas the highest was observed in Varuna (Indian mustard). In canola cultivars, TT cultivars had a higher NHI than non-TT cultivars in 2011, but they had similar NHI in 2012.

In 2011, the control and 200 kg N ha^−1^ treatments had the highest NHI but NHI with 200 kg N ha^−1^ did not differ from the treatment that had a total 100 kg N ha^−1^ in three equal splits at rosette, flowering, and pod development stages (**Table 6**). All treatments with an application of total 100 kg N ha^−1^ had similar NHI. In 2012, higher NHI was observed in the treatments with a single application of 100 kg N ha^−1^ at rosette stage and at flowering stage than the application of 200 and 100 kg N ha^−1^ in five splits at key growth stages, and a single application 100 kg N ha^−1^ at green-bud stage (**Table 7**), but all N treatment had similar NHI compared to control.

#### Nitrogen Use Efficiency

In the higher rainfall season of 2011, N uptake efficiency and N use efficiency of the canola and mustard were similar (**Table 8**). Among the canola cultivars, TT cultivars had lower N uptake efficiency and N use efficiency than non-TT cultivars. In the drier conditions in 2012, mustard had lower N uptake efficiency and NUE than canola. All canola cultivars had similar N uptake efficiency, but TT cultivars had lower N use efficiency than non-TT cultivars.

**Table 8:** Nitrogen efficiencies of different cultivars of canola and mustard during 2011 and 2012.

Cultivars	N uptake efficiency Kg N kg^−1^ N supply		NUE_SY_		Agronomic efficiency kg kg^−1^ N fert.		Physiological efficiency kg kg ^-1^		Apparent recovery %	
	2011	2012	2011	2012	2011	2012	2011	2012	2011	2012
AV-Garnet	1.10^a^	0.35^a^	13.6^a^	5.9^a^	5.3^a^	0.7^b^	34^a^	27^a^	68^a^	22^a^
Fighter TT	0.82^a^	0.29^ab^	9.7^ab^	3.6^bc^	3.7^a^	1.6^ab^	17^a^	43^a^	54^a^	23^a^
Hyola555TT	0.82^a^	0.36^a^	9.2^b^	4.7^ab^	6.2^a^	0.6^b^	23^a^	31^a^	68^a^	8^a^
Hyola575cl	1.16^a^	0.34^a^	12.4^ab^	5.2^a^	4.2 ^a^	1.5^ab^	34^a^	38^a^	58^a^	9^a^
Oasis	1.03^a^	0.22^b^	11.3^ab^	3.1^c^	4.7^a^	3.0^ab^	23^a^	47^a^	56^a^	23^a^
Varuna	1.11^a^	0.23^b^	11.4^ab^	3.1^c^	4.4^a^	4.2^a^	30^a^	46^a^	59^a^	26^a^
Orthogonal comparisons										
Canola vs Mustard	NS	<.0001	NS	<.0001	NS	0.001	NS	NS	NS	0.048
TT vs nonTT	0.0005	NS	0.0004	<.0001	NS	NS	NS	NS	NS	NS
OPc vs HYc	NS	0.0483	NS	NS	NS	NS	NS	NS	NS	0.018

Values are presented as mean of replications and mean of N treatments as there was no cultivar x N treatments interactions were found. Contrast were determined by Tukey test. Different lowercase letters indicate significant difference (Tukey's test P < 0.05). The significance of single degree of freedom orthogonal comparisons are also shown: canola vs mustard (C vs M), TT canola vs non-TT canola (TT vs non-TT), and open pollinated canola vs hybrid canola (OPc vs HYc). A lack of differences between single degree of freedom orthogonal comparisons is noted by NS (not significant).

Nitrogen uptake efficiency was significantly reduced with applications of N in 2011 (**Table 6**) but was similar in all treatments during 2012 when uptake efficiency was lower than 2011 (**Table 7**). Nitrogen use efficiency was significantly reduced with the application of N in both years. Nitrogen uptake efficiency and N use efficiency were not affected by the N rate in both years. The N uptake efficiency was not affected by delaying the N applications in both years, but N use efficiency declined with delayed N application beyond the rosette stage in drier condition of 2012.

Agronomic efficiency varied between the contrasting seasons. In a season with dry post-flowering period (2012), agronomic efficiency was less than half that observed in 2011 (**Tables 6**–**8**). Agronomic efficiency was very similar across canola and mustard cultivars in 2011, but mustard had higher agronomic efficiency than canola during the dry growing conditions of 2012. Orthogonal comparisons of N treatments revealed that agronomic efficiency was higher when N was applied at the rosette growth stage than delaying N until green-bud or flowering in both years. Agronomic efficiency was higher at the green-bud stage than at flowering when N was applied at 100 kg N ha^−1^ in two splits. All other treatments were statistically similar to each other in both years.

There was a strong effect of seasonal conditions on average apparent recovery, being 60% in 2011 and 19% in 2012. In 2011, apparent recovery among the cultivars ranged from 55% to 68%. In 2012, canola had a lower apparent recovery than mustard, possibly due to the lower apparent recovery of hybrid canola than open-pollinated cultivars (**Table 8**). In 2011, delayed application of N to flowering reduced the apparent recovery more than N application at the green-bud stage (**Table 5**) but was similar in 2012 (**Table 6**). All other treatments were similar to each other in both years.

Interestingly, physiological efficiency did not differ much between years, unlike the agronomic efficiency and apparent recovery, which varied considerably between the two years. Average physiological efficiencies were 27 kg kg^−1^ in 2011 and 39 kg kg^−1^ in 2012. In 2012, higher physiological efficiency was observed when a total 100 kg N ha^−1^ was applied in three splits starting at the rosette stage than N applications with a similar amount in two splits at later growth stages. The amount of N applied had no effect on the physiological efficiency of canola and mustard in both in both years. These results indicate that early N application at rosette stage is better than late applications for improving N efficiency.

## Discussion

Nitrogen use efficiency depends on the ability of crops to utilize soil N and the efficiency by with which the N taken up is used for growth and yield formation. This study suggested that N uptake was the more important factor influencing NUE in canola and mustard because uptake efficiency and fertilizer N recovery varied more between seasons and among treatments than physiological efficiency. The results also highlighted the importance of water availability in rainfed environments to the expression of NUE.

The responses to N and the values for NUE among *Brassica* spp. and cultivars varied to a considerable degree with the availability of water during the growing season. Nitrogen use efficiency in 2012 was less than half the values measured in 2011 and this reflected the recovery and uptake of N more so than the physiological efficiency. The timing of N supply and the amount of N uptake are important in determining the uptake and redistribution of N and are important factors affecting a crop's ability to use available N efficiently. In *Brassica* spp. N uptake is most rapid between the rosette and flowering stages (Hocking and Stapper, 2001b) and so maximizing N uptake during this period can make a substantial contribution to improving crop NUE. Yau and Thurling (1987) found that high N uptake was associated with vigorous root growth, which is often related to water availability because N uptake is a function of plant available water (Campbell et al., 2007). A relationship between long and vigorous root growth with higher N uptake has been reported previously (Kamh et al., 2005). The responses to N were greater in 2011 when soil moisture availability was higher due to the greater amount of soil moisture at the start of the season and the higher seasonal rainfall. Similar results were also found by Norton and Wachsmann (2006). They also showed that small changes in crop water use had a substantial effect on the improvement in seed yield. Even though WUE_SY_ did not differ greatly between the two growing seasons, the NUE in the drier season (2012) was only one-third of that observed in the year with average rainfall (2011), indicating the NUE is perhaps more sensitive to seasonal conditions that WUE. It is clear that strategies to improve NUE in rainfed environments, either from N management or genetic improvement, need to consider the effect of moisture availability on N uptake and use.

Previous studies have shown the importance of N on crop water use and vice-versa (Sadras, 2004; Norton and Wachsmann, 2006; Sinclair and Rufty, 2012); effective use of soil moisture and high WUE depends on having adequate supplies of N but equally, responses to high soil N require adequate supplies of soil moisture. This interaction between N and water is described as co-limitation (Sadras, 2004). Riar et al. (2016) examined the degree of N and water co-limitation in canola and mustard and showed that the yield gap between actual and potential yield was reduced in rainfed environments when water and N equally co-limited growth. The interaction between water and N was observed in the two growing seasons. Applying N increased crop WUE but in 2012 when soil moisture was lower, the yield response to N were less (Riar et al., 2017) and N use efficiency was low. Improving the NUE of canola in cropping systems therefore should consider strategies that optimize the WUE and NUE rather than improving NUE alone.

The strong link between WUE and NUE means that soil properties that impede the effective use of soil moisture may have an effect on NUE. Many of the soils of the region have chemical and physical constraints that can limit root growth (Adcock et al., 2007) and the soils in these experiments had high concentrations of boron and increased salinity at depth. However this did not appear to affect water use adversely and N was the major influence on water use. The depth of water use was not affected by N rate. However the amount of water use to increase with N rate, especially in 2011. Norton and Wachsmann (2006) also found that water use by canola and mustard cultivars was influenced by N rate in the Victorian Wimmera.

Apart from water, factors like crop cultivars (Grami and LaCroix, 1977; Yau and Thurling, 1987; Balint and Rengel, 2011) and N supply (Smith et al., 1988; Mason and Brennan, 1998) can contribute to plant growth, grain yield, and N uptake. The early flowering period is critical to yield because seed number per m^2^, a major determinant of yield, is largely determined during this period (Dreccer et al., 2000); therefore, it is important to have adequate uptake of N by this stage. Nitrogen use efficiency (NUE_SY_) is related to the recovery of N by the crop and the degree of remobilization of N during pod formation and seed development. Seasonal conditions had a marked effect on both of these and the pattern of N uptake influenced the degree of N remobilization. Canola is an indeterminate crop and has the potential to maintain N uptake to help meet the demands of the developing seed when there is sufficient soil moisture and this extended period of uptake can enhance N recovery and NUE. This was clearly seen in 2011 when total N uptake was 2.7 times higher than in the drier year, 2012, but N uptake at flowering was only about 20% higher than in 2012. Norton (2016) suggested that canola has the capacity to recover from early stress because N uptake can continue up to the seed filling stage under suitable conditions like high water availability but if the season is dry the ability to recover from early N stress is limited. The differences in post flowering N uptake in the present study support this argument. Most of the total N uptake occurred in the pre-flowering, but post-flowering uptake was still important to yield and NUE. An extended period of N uptake in 2011 improved the N uptake efficiency, N use efficiency, agronomic efficiency and apparent N recovery by between 2.5 and 3.5 fold compared to 2012. It has been suggested that at high N rates yield of canola becomes increasingly source-limited (Dreccer et al., 2000) so the high post-flowering N uptake in 2011 would have also helped maintain green leaf area and photosynthesis rate; a consequence of the continued uptake would be reduced N remobilization from the leaves resulting in low NHI. In contrast, the low soil moisture in 2012 led to low post flowering N uptake, reduced N recovery and increased N remobilization as indicated by higher NHI and increased leaf senescence. Across the two seasons, NHI decreased with increased post-flowering N uptake (r = −0.75; P = 0.0006; n = 11). Nitrogen harvest index values reported here are within the range reported for canola by Papantoniou et al. (2013) [0.25–0.47 in the present study *vs* 0.32–0.66 reported by Papantoniou et al. (2013)] and close to the lower range reported by Ma and Herath (2016) (0.41–0.83) but much lower than the findings of Hocking and Stapper (2001a).

Nitrogen uptake efficiency of cultivars under different N regimes is an important source of variation for NUE (Grami and LaCroix, 1977; Yau and Thurling, 1987; Möllers et al., 1999; Zhang et al., 2010). However there were no cultivar × nitrogen interactions in this study for N uptake efficiency and other NUE parameters indicating that the genetic differences were consistent across different N treatments. Earlier studies have also reported no significant interactions between N and cultivars (Norton, 2002; Riffkin et al., 2012; Riar et al., 2014). This is an important result because it suggests that the genetic differences in NUE were associated with the characteristics of growth among the varieties rather than being affected by the amount and pattern of N supply. It also suggests that selecting for high NUE for different background levels of N may be feasible.

Nevertheless, there were consistent differences in productivity among the cultivars. The differences in WUE and NUE_SY_ between the TT and non-TT cultivars were stable and consistent with earlier studies (Robertson and Kirkegaard, 2006). The cultivars used in this study had similar stable carbon isotope discriminations, indicating they had similar intrinsic transpiration efficiencies. This suggests the lower radiation use efficiency of TT cultivars (Robertson et al., 2002) may be the reason for their low WUE_SY_ and WUE_DM_ compared to the non- TT cultivars. Water use efficiency for seed yield values for canola and mustard cultivars reported here are within the range of 3–18 kg ha^−1^ mm^−1^ reported from 42 different case studies simulated by Robertson and Kirkegaard (2006). The poorer growth of the TT-cultivars also influenced their NUE and N recovery. In 2011, N uptake by the TT canola was lower than non-TT cultivars even though their crop water use was similar. Post-flowering N uptake was also higher in non-TT cultivars, which could be related to their more vigorous shoot and root growth during early growth as water extraction depth for TT and non-TT cultivars at maturity was similar. Use of the TT cultivars has improved the options available for weed management in cropping systems, but the inherently low RUE of these cultivars (Robertson et al., 2002) is a constraint to improved NUE.

Mustard was compared to canola because it is considered more suited to low rainfall environments, partly because of its early flowering, but in the present experiments it did not show a consistent difference in NUE compared with canola. Nitrogen uptake efficiency of canola and mustard was similar in a year with average rainfall (2011), but mustard had lower N uptake efficiency and NUE_SY_ than canola in the drier conditions of 2012, primarily due to its lower yield in 2012 (Riar et al., 2017). Mustard used less water during the pre-flowering phase than canola, which was attributed to its shorter pre-flowering duration. On average over the 2 years, the pre-flowering period of the canola cultivars was 15 d longer than the mustard varieties (93 *vs* 79 *d*). Therefore, a higher proportion of water use in mustard was associated with dry matter accumulation during the post-flowering period. Thurling (1974) also found that mustard produced 85% of its dry matter during the post-flowering growth, whereas canola produced 55% of its total dry matter during this phase. Physiological efficiency of canola and mustard did not differ between 2011 and 2012, but the poorer N uptake efficiency in 2012 indicates that the differences in NUE_SY_ between the 2 years were related to the recovery of N. The shorter pre-flowering period, when N uptake is at its peak (Hocking and Stapper, 2001b), may have limited N uptake despite a longer post-flowering period. Crop phenology is an important characteristic that affects yield and NUE, but there has been relatively little work to look at the importance of phenology to N recovery and NUE in canola.

Among the efficiency parameters, physiological efficiency showed relatively little variation between seasons and treatments. The values for physiological efficiency (27 kg kg^−1^ in 2011 and 39 kg kg^−1^ and 2012) are similar to those reported by Smith et al. (1988) in rapeseed and by Anderson and Hoyle (1999) for wheat for these Mediterranean environments. The relative consistency in physiological efficiency among treatments and the similarities between different experiments suggests that there may be limited variation in this trait and that a focus for future work should be on recovery of soil and fertilizer N.

Targeting N application to specific phenological growth stages altered the pattern of crop water use, which influenced N uptake and NUE of canola and mustard in this Mediterranean environment. The timing of N application at different growth stages did not influence the total N uptake at maturity, which was affected more by the rate of N supply in both years. Similar findings were reported by Marquard and Walker (1995). However, targeting early applications of N (rosette, green bud, and flowering stages), when the rate of N uptake was increasing, improved early N uptake and enhanced NUE parameters. In these trials, it was also found that greatest yield responses were with N applied at the rosette stage (Riar et al., 2017). These responses can be driven from the association of N with water as this period of vegetative growth of canola and mustard corresponded with high water availability in both years. Higher rates of N application and delayed N application beyond the rosette stage decreased the agronomic efficiency of canola and mustard in both years.

For future plant based research to improve NUE, one should consider the effect of cultivars with different degrees of vigor to N uptake. In this study cultivars (non-TT and hybrids) with high early vigor had high N uptake, which could also indicate their root vigor as high N uptake with vigorous roots was reported by Yau and Thurling (1987). This can solve the problem of N uptake from soil but not internal N utilization. Even though we did not find any variation in physiological efficiency for the cultivars studied, Svečnjak and Rengel (2006) found variation in internal N utilization of different canola cultivars and this variation can be utilized to find cultivars more efficient in acquired N remobilization. Genes associated with enzyme alanine aminotransferase have been already incorporated in to Canadian canola cultivars (Norton, 2007) this can help crop to have high N remobilization under stress conditions. The present study suggests that improvements in NUE were achieved by increasing the recovery of N rather that substantial changes in the physiological efficiency. Therefore, crops traits and management practices that will increase N recovery should be a focus of future work. In these environments, early uptake of N is important because crop yields are most responsive to N applied at the rosette-green bud stage, but the ability to maintain N uptake during the post-flowering period when soil moisture availability is high will also improve NUE. To improve N recovery and NUE_SY_ applying N at early growth stage, prior to the start of flowering, and exploring the genetic potential of canola and mustard cultivars to take up N from soil should be the focus of future work.

## Conclusions

This study highlights the importance of N uptake and water availability to the expression of NUE. N responses and values of NUE varied considerable with water availability during growing season. Seasonal conditions affected N uptake and influenced the degree of N remobilization. Most of the total N uptake occurred in the pre-flowering, but post-flowering uptake was still important to yield and NUE. Post flowering N uptake in the present study clearly showed the capacity of canola and mustard to recover from early stress. In these environments, NUE appears to be more sensitive to seasonal conditions than WUE indicating that clear strategies to improve NUE in rainfed environments need to consider the effect of moisture availability on N uptake and use from N management or genetic improvement. Water and N interaction of this study also highlight the need of strategies that optimize the WUE and NUE rather than improving NUE alone. Genetic differences in NUE were associated with the characteristics of growth among the varieties rather than being affected by the amount and pattern of N supply. It also suggests that selecting for high NUE for different background levels of N may be feasible. Shorter pre-flowering period of mustard limited pre-flowering N uptake and future work should look at the importance of phenology to N recovery and NUE in canola and mustard. For future plant based research to improve NUE, one should consider the effect of cultivars with different degrees of vigor to N uptake. The relatively consistent physiological efficiency or relative consistency physiological efficiency or relative consistency in physiological efficiency among treatments and the similarities between different experiments suggests that there may be limited variation in this trait and that a focus for future work should be on recovery of soil and fertilizer N.

## Data Availability Statement

All datasets presented in this study are included in the article/**Supplementary Material**.

## Author Contributions

Conceptualization and methodology: AR, GM, and GG. Investigation: AR. Validation and formal analysis: AR and GM. Data curation: AR. Writing—original draft preparation: AR. Writing—review and editing: AR, GM, and GG. Visualization: AR. Supervision: GM and GG. Project administration: AR and GM. Funding acquisition: AR. All authors contributed to the article and approved the submitted version.

## Funding

This research was funded by John Allwright Fellowship from the Australian Centre for International Agricultural Research (ACIAR). Grant number: S00052W9.

## Conflict of Interest

The authors declare that the research was conducted in the absence of any commercial or financial relationships that could be construed as a potential conflict of interest.
