# Supplementary material for: Different Post-Sowing Nitrogen Management Approaches Required to Improve Nitrogen and Water Use Efficiency of Canola and Mustard
Source: Front Plant Sci. 2020 Jul 21;11:1111. doi: 10.3389/fpls.2020.01111 (PMC7385279; doi:10.3389/fpls.2020.01111)
Supplement: Supplementary file 1 [file Table_1.docx]

## Supplementary Material

Table S1: Carbon isotope discrimination of Canola and Mustard cultivars grown under rainifed conditions in 2011 at Roseworthy. Carbon isotope discrimination was measured on leaf samples taken at early- to mid-flowering.

| Specie Type | Cultivar ! | Cultivar Type | | | Δ ‰ (Mean ± s.e) | Δ ^13^C PDB (Mean ± s.e) |
| --- | --- | --- | --- | --- | --- | --- |
| Canola | AV Garnet | Open-pollinated | | | 19.33 ± 0.78 | -26.80 ± 0.74 |
| Canola | FighterTT | | | Open-pollinated | 18.74 ± 0.29 | -26.24 ± 0.28 |
| Canola | Hyola 555TT | | Hybrid | | 19.79 ± 0.50 | -27.25 ± 0.48 |
| Canola | Hyola 575cl | | Hybrid | | 19.34 ± 0.13 | -26.82 ± 0.12 |
| Mustard | Oasis | Open-pollinated | | | 19.77 ± 0.39 | -27.23 ± 0.37 |
| Mustard | Varnua | | Open-pollinated | | 19.22 ± 0.61 | -26.70 ± 0.58 |
